# Genome-wide identification and expression analysis of JmjC domain-containing family in *Vitis vinifera* L.

**DOI:** 10.1186/s12864-025-11995-y

**Published:** 2025-09-26

**Authors:** Mengqi Wang, Jie Wang, Wangsheng Zhu, Xin Sun

**Affiliations:** School of Environment and Tourism, West Anhui University, Lu’an, 237012 China

**Keywords:** *Vitis vinifera*, JMJ, Gene family, Gene expression, Transcriptional profiling analysis

## Abstract

**Background:**

In eukaryotes, histone methylation is essential for controlling chromatin structure, gene transcription, and related chromatin processes. Jumonji C (JmjC) domain-containing demethylases (JMJs) regulate histone methylation levels. The *JMJ* gene family has been discovered in various plants, including *Arabidopsis thaliana*, *Oryza sativa*, *Malus domestica*, and *Zea mays*.

**Results:**

In this study, a total of 23 *Vitis vinifera VvJMJ* gene family members related to histone methylation were identified utilizing genomic and transcript databases. We analyzed the chromosomal location distribution, protein properties, phylogenetic relationships, gene structures, motifs, evolutionary patterns, promoter sequences, and expression profiles of the *VvJMJ* gene family. The *VvJMJ* genes were classified into five subfamilies: KDM3, KDM4, KDM5, JMJD6, and JMJC. Co-linearity analysis showed that *VvJMJs* are highly conserved with *AtJMJs* and *OsJMJs* genes. Analysis of the promoter sequences revealed that *VvJMJ* gene promoter regions are enriched with elements associated with stress response, growth and development, hormone response, and light reactions. Transcriptional profiling and qRT-PCR analysis showed that *VvJMJs* may play various roles in the multiple biological processes of *V. vinifera*. For example, *VvJMJ2*, *VvJMJ16*, *VvJMJ18* may participate in the ripening process of *V. vinifera* fruits; *VvJMJ1*, *VvJMJ4*, *VvJMJ6*, *VvJMJ13*, *VvJMJ17*, *VvJMJ20*, *VvJMJ21, VvJMJ23* may participate in various hormone responses; *VvJMJ1*, *VvJMJ4*, *VvJMJ10*, *VvJMJ11* may participate in the stress response of *V. vinifera*.

**Conclusions:**

This study initially identified the characteristics of *V. vinifera* JmjC domain-containing genes. The results established a basis for functional analyses of *VvJMJ* genes in *V. vinifera*, facilitating the exploration of the mechanisms of underlying the growth, development, and fruit maturation.

**Supplementary Information:**

The online version contains supplementary material available at 10.1186/s12864-025-11995-y.

## Introduction

Recent advancements in histone modification detection technologies and related research have revealed that histones undergo post-translational modifications not only at the N-terminus amino acid residues but also at the middle and C-terminal regions [1]. These modifications influence gene expression and regulate various growth and developmental processes in organisms [2]. Histone modifications encompass methylation, acetylation, phosphorylation, ubiquitination, among other types [3]. Histone methylation is a reversible and dynamic regulatory process, including methylation and demethylation. The occurrence, removal, and functional roles of these modifications are primarily regulated by histone modification enzymes and their cofactors, known as Writers, Erasers, and Reader/Effectors [4]. The steady-state equilibrium of histone methylation is dynamically regulated by two counteracting enzyme families: histone methyltransferases (HMTs) and histone demethylases (HDMs). These enzymes achieve this regulation through post-translational modifications, either depositing or removing methyl groups from specific histone residues, thereby controlling transcription factor accessibility to genomic DNA [5]. Histone demethylases, characterized by the JmjC domain, are crucial for maintaining histone methylation homeostasis and are significantly linked to plant growth and development. Research has identified proteins encoding JMJ-C genes in *Arabidopsis thaliana* [6], *Zea mays* [7], *Citrus sinensis* [8], *Oryza sativa* [9] *Gossypium hirsutum* [10], *Solanum lycopersicum* [11], *Malus domestica* [12], and *Jatropha curcas* [13].

In *Arabidopsis*, 21 JmjC proteins are categorized into five subfamilies based on sequence similarity: KDM4/JHDM3, KDM5/JARID1, JMJD6, KDM3/JHDM2, and JMJ-C only domain [6]. They are crucial in controlling leaf development, floral transition, flowering timing, and response to abiotic stress [14]. *AtJMJ16*, *AtJMJ14*, *AtJMJ15*, *AtJMJ18*, *AtJMJ12*, and *AtJMJ13* regulate plant development by modulating histone methylation. *AtJMJ16* reduces H3K4me3 levels to inhibit leaf maturation and regulate senescence [15]. *AtJMJ14* decreases H3K4me to suppress *FT* expression, promoting floral transition [16]. *AtJMJ15* and *AtJMJ18* lower H3K4 levels to reduce *FLC* expression, stimulating flowering [17]. *AtJMJ12* and *AtJMJ13* adjust FLC histone methylation in response to temperature and light, influencing flowering time in *Arabidopsis* [18]. In *O. sativa*, 20 proteins containing the JmjC domain have been identified [6, 19]. *OsJMJ706* functions as an H3K4me 1/2/3 demethylase and plays a role in regulating the development of floral organs in *O. sativa* [20]. OsJMJ703, an H3K4 demethylase, is essential for *O. sativa* transposon silencing, stem elongation, and drought stress defense [21–23]. In *Medicago truncatula*, *MtJMJC5* experiences cold-induced alternative splicing and potentially plays a role in freezing tolerance response [24]. Moreover, in many higher plants, including *Solanum lycopersicum* and *Musa nana*, fruit development is also closely related to histone methylation. *Solanum lycopersicum SlJMJ6* demethylates fruit maturation-related genes and H3K27me3, activating *ACS4* and *ACO1* to enhance mature inhibitor expression [11]. Meanwhile, *S. lycopersicum SIJMJ7* demethylates H3K4me1, H3K4me2, and H3K4me3, directly suppressing *DMS2* expression to regulate fruit maturation [25]. *Musa nana MaJMJ15* facilitates fruit maturation by demethylating H3K27me3 on chromatin, thereby activating key RRGs expression [26].

Vitis viniferas, a woody vine from the *Vitis vinifera* family, are globally prevalent and valued for their economic, nutritional, and medicinal benefits [27]. Research on *V. vinifera* histone demethylase gene identification and function are currently limited. This study employed bioinformatics to systematically identify and characterize the *V. vinifera VvJMJ* gene family. Comprehensive analyses were subsequently performed to investigate their physicochemical properties, chromosomal distribution, phylogenetic relationships, gene evolution, motifs, conserved domains, and *cis*-elements. Additionally, we analyzed the types of gene duplication within the *VvJMJ* gene family and confirmed that its expansion primarily relies on transposed duplication. Utilizing transcriptome data, we systematically investigated the performance of *VvJMJ* family members across varietal differences, fruit development, and responses to biotic and abiotic stresses. This multidimensional analysis enhanced the depth and breadth of existing research. This detailed study of *VvJMJ* genes establishes a basis for future research into their roles in *V. vinifera* growth, development, stress response, and hormone signaling. These results will provide valuable genomic insights and directions for further research on the *VvJMJ* genes.

## Materials and methods

### Identification and classification of *V. vinifera JMJ* genes

The *V. vinifera* genome and gff annotation files were obtained from the Ensemble plant online website (https://plants.ensembl.org/). The *Arabidopsis JMJ* gene family amino acid (aa) sequences were obtained from the TAIR (https://www.arabidopsis.org/). The gene identifier (ID) is available in Table S1.

Candidate *JMJs* genes in *V. vinifera* were identified using two BLAST methods implemented in TBtools [28]. BLAST analysis was conducted using the protein sequences of *Arabidopsis* JMJs as queries. Additionally, relevant hidden Markov models (PF02373) were obtained from the Pfam database (http://pfam.xfam.org/), and employed to search the *V. vinifera* protein sequence data using the HMMER 3.0 software with a stringent threshold of E ≤ 10^–20^. A preliminary set of candidate sequences was determined after eliminating redundancy and duplicates. The gene family candidate members were identified by integrating the results from the aforementioned methods. The Web CD-Search tool on the National Center for Biotechnology Information (NCBI) website (https://www.ncbi.nlm.nih.gov/Structure/bwrb/bwrpsb.cgi/) was utilized to analyze the conserved protein domains of the target gene family in *V. vinifera* [29]. This study investigated the presence of the conserved domain associated with the target gene family protein in each candidate sequence. Candidate sequences with complete domains were selected for further analysis. Sequences with varying or incomplete domains were first evaluated for integrity using the SoftBerry website (http://linux1.softberry.com/), followed by analysis with the Batch Web CD-Search tool to compare their domain similarity with *Arabidopsis* JMJs sequences.

The physicochemical properties of gene family proteins were assessed using ExPASy ProtParam tool (https://web.expasy.org/protparam/), which includes parameters like aa count, molecular weight, isoelectric point (pI), instability index, aliphatic index, and grand average of hydropathicity (GRAVY). The chromosomal locations of confirmed histone methylation modification genes were extracted from the *V. vinifera* genome's GFF3 file and visualized on *V. vinifera* chromosomes using TBtools. The subcellular localization of *V. vinifera JMJ* genes was predicted using the Wolf Psort online tool (https://wolfpsort.hgc.jp/), with the most likely prediction chosen as the result.

### Phylogenetic analysis of histone methylation modification proteins in *V. vinifera* and *A. thaliana*

Amino acid sequences of *JMJs* in *Arabidopsis* and *V. vinifera* were sourced from the TAIR and Ensemble plant databases, respectively. The sequences were aligned with MUSCLE in MEGA 11. The optimal model was selected using MEGA 11 software, and a phylogenetic tree was constructed via the maximum likelihood (ML) method [30]. The evolutionary history of the analyzed taxa is represented by the bootstrap consensus tree derived from 1000 replicates [31]. TBtools was used to visualize and optimize the phylogenetic tree.

### Chromosomal distribution and synteny analysis

The Amazing Gene Location tool in TBtools was utilized to map JMJs to chromosomes using data from GTF/GFF files. The Dual Synteny Plotter tool in TBtools was used to visualize synteny blocks within the *V. vinifera* genome and between the genomes of *V. vinifera*, *Arabidopsis*, and *O. sativa*. The One Step MCScanX Super Fast method was used to generate these synteny blocks. The *O. sativa* genome data were obtained from the Ensembl Plants database (https://plants.ensembl.org/index.html).

### Identification of duplicated gene types

We employed the DupGen_finder tool (https://github.com/qiao-xin/DupGen_finder) to classify the types of duplicated genes. DupGen_finder_unique categorizes plant genome duplicated genes into five categories based on a specific algorithm: whole-genome duplication (WGD), proximal duplication (PD), transposed duplication (TRD), dispersed duplication (DSD), and tandem duplication (TD) [32]. Using *Arabidopsis* as an outgroup for *V. vinifera*, we followed the recommended workflowof DupGen_finder for our analysis and parameter configuration.

### Sequence analysis

The conserved features of JMJs sequences were analyzed and visualized based on motifs using the Multiple Em for Motif Elicitation (MEME) suite 5.4.1 (https://meme-suite.org/meme/tools/meme) [33] and TBtools. The gene structure of *JMJ* genes were assessed using Gene Structure Shower in TBtools, utilizing information from the *V. vinifera* genome GFF3 file. To identify the *cis*-elements in the promoters of *JMJ* genes, PlantCare (https://bioinformatics.psb.ugent.be/webtools/plantcare/html/) was employed [34].

### Expression profiling of *JMJ* genes in *V. vinifera*

*JMJ* gene expression patterns under abiotic, hormone, and biotic stress treatments were retrieved from the SRA database on the NCBI website. The study IDs included *V. vinifera* fruit development (GSE62745, GSE62744), NAA and ABA response (GSE150343), MeJA and SA response (PRJNA845078), light stress (GSE129916), and *Colletotrichum viniferum* stress (PRJNA952825). The expression of genes was quantified using Kallisto, an RNA-seq analysis tool [35]. This study estimated gene expression using the FPKM (fragments per kilobase of exon per million mapped reads) method.

### Plant materials and hormone treatment

The experimental material used was six year old *V. vinifera* cv.'Muscat Hamburg'. One week before the fruit color change period, a solution of 500 mg/L ethylene (ETH) containing 0.05% Tween 20 was sprayed on the fruit. The control treatment (CK) involved spraying the fruit with distilled water containing 0.05% Tween 20. The experiment was repeated three times, with each treatment consisting of three plants. Sampling was conducted at 0 d, 5 d, 10 d, and 15 d after treatment. For each replicate, 10 bunches of fruit were randomly sampled, deseeded, frozen in liquid nitrogen, and stored at −80 °C.

### RNA extraction, library construction, sequencing, and data analysis

Total RNA was extracted from berry skins using the RNAprep Pure Plant Kit (TianGen, Beijing, China) in accordance with the manufacturer's instructions. The integrity of the extracted RNA was confirmed through gel electrophoresis, while the concentration and quality were assessed using a NanoDrop One Spectrophotometer (Thermo Scientific, MA, USA). A total of 3 μg of RNA was utilized to construct an RNA-Seq library with the NEBNext® Ultra™ RNA Library Prep Kit for Illumina® (NEB, USA). Subsequently, all library preparations were sequenced on an Illumina HiSeq platform (HiSeq 4000, 150PE) by Novogene Bioinformatics Technology Co. Ltd (Beijing, China).

Raw data in FASTQ format were initially processed using in-house Perl scripts by Novogene (China). Clean reads were obtained by removing adaptors and low-quality read pairs. The Vitis vinifera gene sequence and annotation for ‘Pinot noir’ (12X) were downloaded from http://www.genoscope.cns.fr/externe/GenomeBrowser/Vitis/. Bowtie v2.2.3 was employed to index the reference genome [36], and HISAT 2.0 was utilized to map clean reads to the grapevine reference genome [37]. HTSeq v0.6.1 was used to calculate the number of reads mapped to each gene [38]. Fragments Per Kilobase Million (FPKM) for each gene were calculated based on gene length and the number of reads mapped to that gene [39]. Differential expression analysis for each group (two biological replicates per group) was performed using the DESeq R package (1.18.0) [39]. Genes with |log2(Fold change)| greater than 1 and FDR ≤ 0.05 were defined as differentially expressed genes (DEGs).

### Quantitative reverse transcription polymerase chain reaction

Total RNA was extracted from *V. vinifera* fruits using the hot borate method as outlined in prior research [40]. First-strand cDNA was synthesized from 1 µg of total RNA using Reverse Transcriptase M-MLV (RNase H-) (Takara Biomedical Technology Co., Ltd., Shiga, Japan) for reverse transcription-PCR. qRT-PCR was performed using Takara SYBR Premix Ex Taq II (Takara Biomedical Technology Co., Ltd.) on a Light Cycler 480 instrument (Roche, Basel, Switzerland)*.* The amplification parameters were 95 ℃ for 3 min followed by 40 cycles at 95 ℃ for 15 s, 60℃ for 30 s, 72 ℃ for 30 s for plate reading. *Ubiquitin1* served as an internal control [41]. The primers utilized are detailed in Supplementary Table S2.

## Results

### Identification of *JMJs* in *V. vinifera*

The *V. vinifera JMJ* gene family was identified by using *Arabidopsis* protein sequences and the *JMJ* gene family structural domain (PF02373) as search queries, resulting in 23 identified members, as detailed in Table 1.

**Table 1:** *JMJ* genes identified in *V. vinifera*

Gene Name	Gene ID	Chromosome	Length (aa)	MW (kDa)	pI	II	AI	GRAVY	Subcellular localization prediction
*VvJMJ1*	Vitvi02g00329	chr2	1852	210383.12	6.27	47.75	91.33	−0.25	Chloroplast
*VvJMJ2*	Vitvi02g00759	chr2	876	99182.32	6.54	56.49	70.25	−0.50	Nuclea
*VvJMJ3*	Vitvi02g04368	chr2	70	7937.22	7.77	14.49	71.14	−0.15	Cytoplasmic
*VvJMJ4*	Vitvi02g01290	chr2	371	41541.88	5.53	58.85	77.84	−0.30	Nuclea
*VvJMJ5*	Vitvi04g01989	chr4	509	57077.70	5.83	55.48	68.92	−0.81	Nuclea
*VvJMJ6*	Vitvi06g00167	chr6	411	45958.13	5.05	53.51	85.91	−0.20	Nuclea
*VvJMJ7*	Vitvi07g00075	chr7	544	61130.22	5.25	50.55	89.10	−0.29	Nuclea
*VvJMJ8*	Vitvi07g00618	chr7	899	102075.98	5.78	55.16	67.60	−0.58	Nuclea
*VvJMJ9*	Vitvi07g04214	chr7	252	27661.12	5.97	51.47	71.23	−0.34	Nuclea
*VvJMJ10*	Vitvi08g00216	chr8	507	57836.18	7.97	49.30	83.20	−0.30	Nuclea
*VvJMJ11*	Vitvi10g00053	chr10	881	100648.82	6.74	59.31	71.77	−0.64	Nuclea
*VvJMJ12*	Vitvi10g00339	chr10	1021	117007.49	8.63	58.72	82.02	−0.53	Nuclea
*VvJMJ13*	Vitvi10g01120	chr10	1086	122284.90	5.31	53.43	72.14	−0.47	Nuclea
*VvJMJ14*	Vitvi10g01394	chr10	1256	139897.99	7.00	52.98	60.60	−0.98	Nuclea
*VvJMJ15*	Vitvi13g04179	chr13	1533	169920.49	6.01	49.18	72.82	−0.58	Nuclea
*VvJMJ16*	Vitvi14g00196	chr14	946	107326.79	5.66	46.86	72.94	−0.72	Nuclea
*VvJMJ17*	Vitvi14g00627	chr14	1271	142180.89	6.08	49.19	76.86	−0.47	Nuclea
*VvJMJ18*	Vitvi15g00950	chr15	1011	114583.91	7.88	47.31	65.58	−0.83	Nuclea
*VvJMJ19*	Vitvi16g00120	chr16	659	74924.90	9.04	45.42	67.75	−0.55	Nuclea
*VvJMJ20*	Vitvi16g01410	chr16	2319	254657.19	5.73	43.61	66.25	−0.74	Nuclea
*VvJMJ21*	Vitvi17g00875	chr17	493	57514.20	5.26	39.53	79.11	−0.41	Chloroplast
*VvJMJ22*	Vitvi17g01001	chr17	1329	148429.94	8.64	49.22	68.10	−0.58	Nuclea
*VvJMJ23*	Vitvi18g00437	chr18	970	110481.76	5.38	44.11	83.33	−0.34	Cytoplasmic

Within the identified family members, *VvJMJ3* was unique with a predicted aa sequence length of 70 aa, whereas the remaining *VvJMJ* family members exhibited aa lengths between 252 aa (*VvJMJ9*) and 2319 aa (*VvJMJ20*). The predicted molecular weight of *VvJMJ3* was 7937.22 Da, while the other members ranged from 27661.12 Da (*VvJMJ9*) to 254657.19 Da (*VvJMJ20*). The predicted pI varies from 5.05 (*VvJMJ6*) to 9.04 (*VvJMJ19*), with 17 genes encoding proteins that are alkaline (PI ≤ 7) and six genes encoding proteins that were acidic. The instability index (II) ranged from 14.49 (*VvJMJ3*) to 59.31 (*VvJMJ11*). Two genes, *VvJMJ3* and *VvJMJ21*, encodes stable proteins with instability coefficients below 40 in vitro. In contrast, proteins encoded by the remaining 21 genes had instability coefficients exceeding 40, indicating in vitro instability. The aliphatic index (AI) of *V. vinifera JMJ* family proteins varies from 60.60 (*VvJMJ14*) to 91.33 (*VvJMJ1*), while the grand average of hydropathicity (GRAVY) ranges from −0.98 (*VvJMJ14*) to −0.15 (*VvJMJ3*), indicating their hydrophilic nature as all GRAVY values are negative. Predicted subcellular localization suggested that while *VvJMJ1* and *VvJMJ21* are in the chloroplast and *VvJMJ3* and *VvJMJ23* are in the cytoplasm, the remaining VvJMJ proteins are nuclear, indicating a primary nuclear function for this gene family.

### Analysis of chromosomal distribution and evolutionary relationships of *VvJMJ* genes

**Fig. 1:**
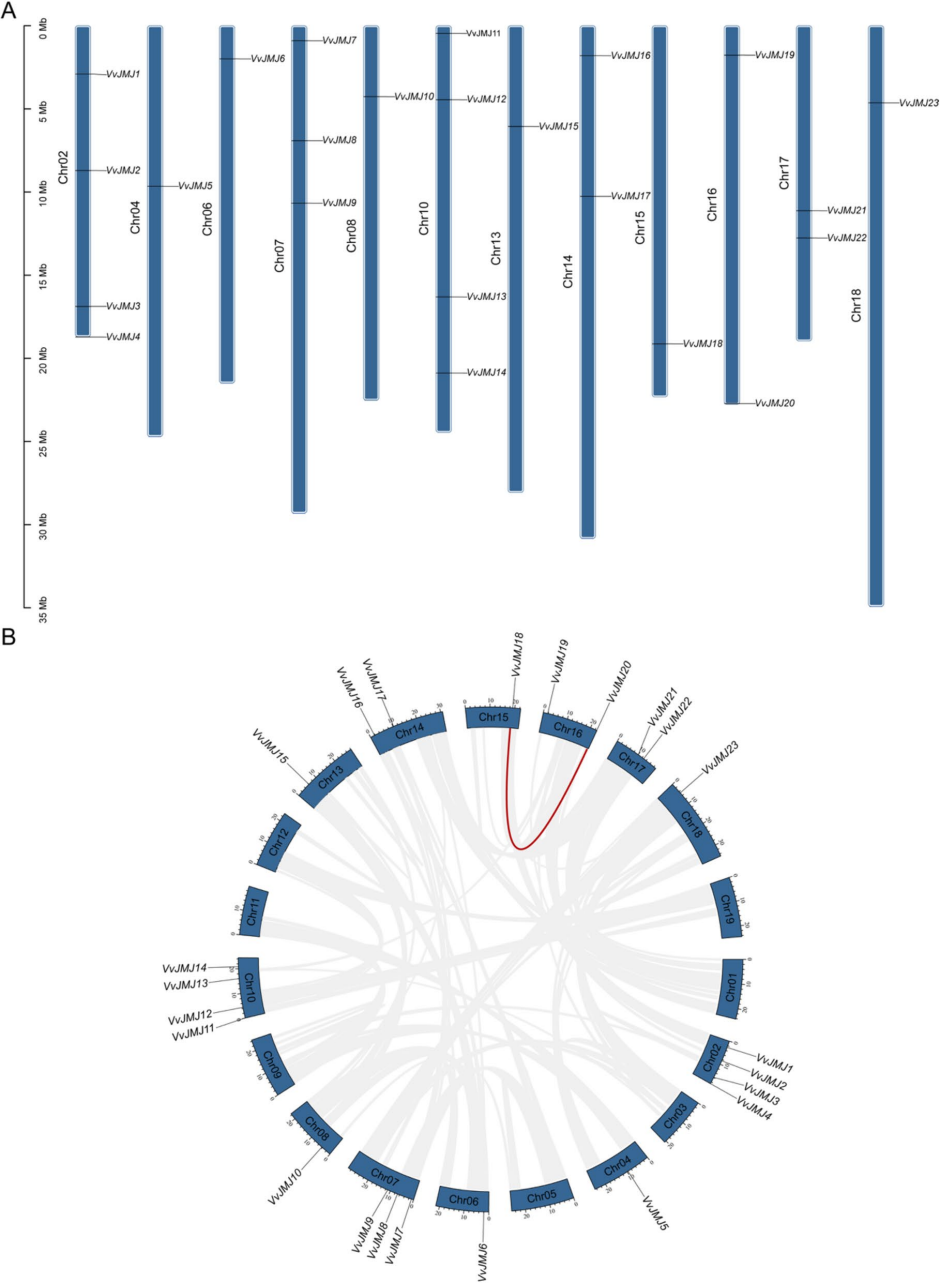
Chromosomal distribution and collinearity analysis of *JMJ* genes in *V. vinifera*. **A** Chromosomal distribution and duplication analysis of *JMJs* in *V. vinifera*. **B** Collinearity analysis of *JMJ* genes family in *V. vinifera*

Figure 1A illustrated the chromosomal distribution of *JMJ* genes in *V. vinifera*. The analysis revealed that the *V. vinifera* genome comprises 18 chromosomes, with *VvJMJ* genes distributed unevenly across 12 of these chromosomes (not present on chromosomes 01, 03, 05, 09, 11, and 12). These genes were named *VvJMJ1* to *VvJMJ23* based on their arrangement along the chromosomes. The distribution pattern of *VvJMJ* genes on the 12 chromosomes was not uniform, with most genes located in the central region of the chromosomes. Only *VvJMJ4* and *VvJMJ20* were found at the distal ends of chromosomes 2 and 16, respectively. Furthermore, chromosomes 2 and 10 each contain four genes, the highest number; chromosome 7 had three genes, the second highest; chromosomes 14, 16, and 17 had two genes each; and chromosomes 4, 6, 8, 13, 15, and 18 had only one gene each, the lowest number. The scattered distribution of *V. vinifera JMJ* gene family members across the 12 chromosomes suggested the possibility of genetic variation during the natural growth and development of *V. vinifera* [25]. The results of the chromosomal positioning did not show any gene tandem duplication phenomena. Figure 1B illustrated that gene co-linearity analysis reveals a pair of genes, *VvJMJ18* and *VvJMJ20*, demonstrating strong co-linearity. This suggested they have experienced segmental duplication and are paralogous. Chromosomal positioning analysis revealed no tandem duplication genes within the *V. vinifera JMJ* gene family. The presence of one pair of genes undergoing segmental duplication and the absence of tandem duplication pairs in the *V. vinifera JMJ* gene family suggested that segmental duplication events contribute to its expansion.

The analysis of gene collinearity and gene duplication events using the MCScanX software revealed the evolutionary relationship of the *V. vinifera JMJ* gene family. Figure 1B illustrated that collinearity analysis identifies a gene pair, *VvJMJ18* and *VvJMJ20*, with strong collinearity, suggesting they are paralogous genes resulting from segmental duplication. However, the chromosomal location analysis did not identify any tandem duplication genes in the *V. vinifera JMJ* gene family. In the *V. vinifera JMJ* gene family, a single gene pair had experienced segmental duplication, indicating that such events contribute to the family's expansion, with no evidence of tandem duplication.

**Fig. 2:**
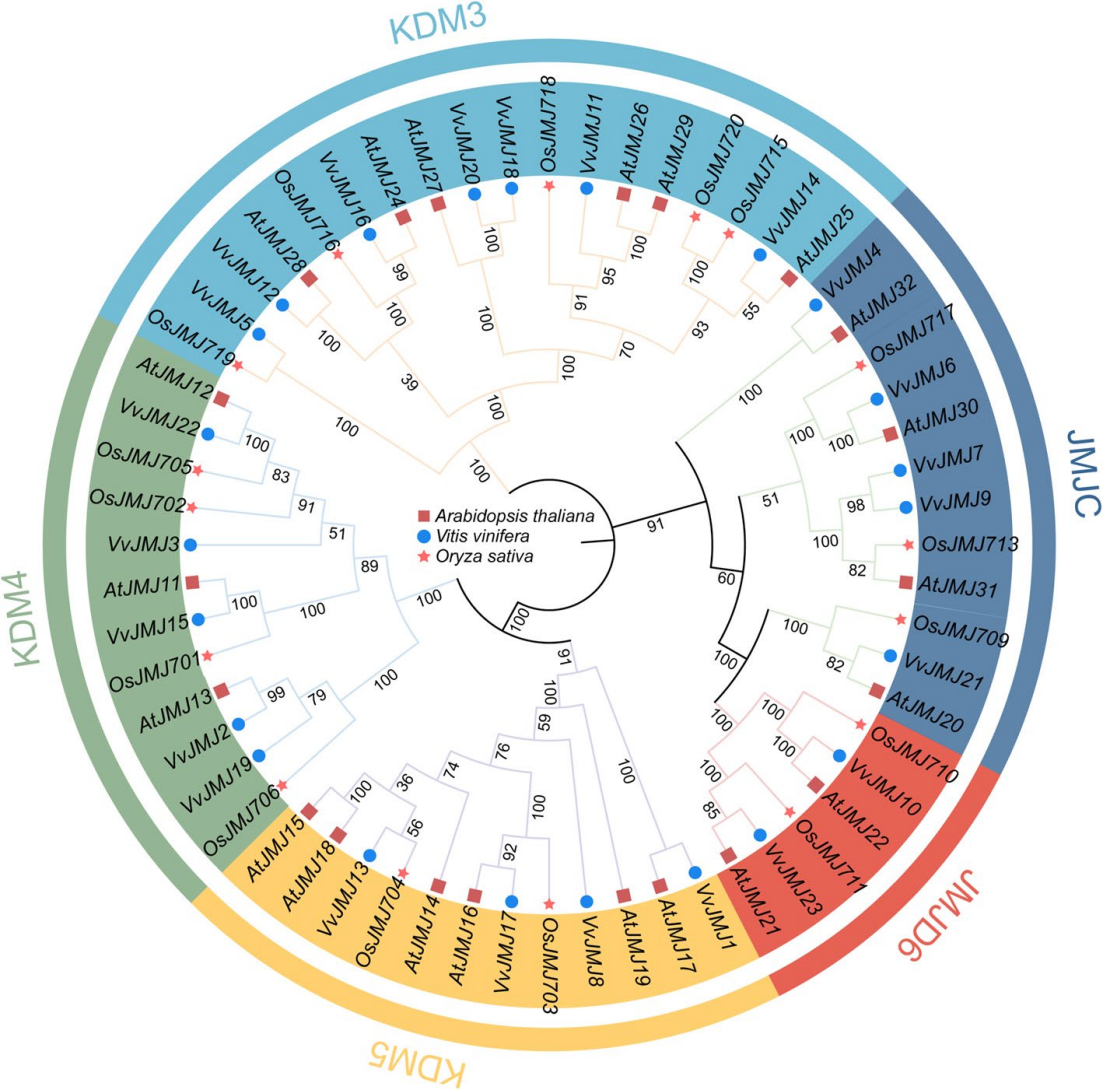
Phylogenetic relationship of JMJ proteins from *V. vinifera*, *O. sativa*, and *Arabidopsis*. The bootstrap consensus of tree was inferred from 1000 replicates, and the phylogenetic tree was constructed using the ML method. In the figure, squares represent *Arabidopsis* proteins, circles represent *V. vinifera* proteins, and stars represent *O. sativa* proteins

To analyze the distribution of subfamily members in the *V. vinifera JMJ* gene family and their phylogenetic relationships with *Arabidopsis* and *O. sativa*, aa sequences from 21 *AtJMJs* and 16 *OsJMJs* genes were extracted from the genomes of these model plants. Clustal was used for multiple sequence alignment, followed by phylogenetic tree construction using the ML method in MEGA11, with a bootstrap value of 1000. iTOL (https://itol.embl.de/) was utilized to visualize and enhance the phylogenetic tree, illustrating the evolutionary relationships within the *V. vinifera JMJ* gene family (Fig. 2). In order to analyze the distribution of the subfamily members of the *V. vinifera JMJ* gene family and their phylogenetic relationships with *Arabidopsis* and *O. sativa*, the aa sequences of 21 *AtJMJs* and 16 *OsJMJs* genes were extracted from the genomes of these model plants to construct phylogenetic trees, and the evolutionary relationship of the *VvJMJ* gene family was elucidated (Fig. 2). Phylogenetic analysis classified 23 V*. vinifera JMJ* genes into five major subfamilies: JARID1/KDM5, JHDM3/KDM4, JHDM2/KDM3, JMJD6, and JmjC domain-only (JmjC), alongside corresponding members from *Arabidopsis* and *O. sativa*. Among the five subfamilies: KDM3 had the most members, with seven *VvJMJs* genes, accounting for 30.4% of the total members; both JMJC and KDM4 subfamilies contained five *VvJMJs* genes, accounting for 21.7%; KDM5 subfamily had four *VvJMJs* genes, accounting for 17.39%; and JMJD6 had only two *VvJMJs* genes, accounting for 8.69%. Most *VvJMJ* genes clustered with *Arabidopsis* genes, suggesting a closer genetic relationship between *V. vinifera* and *Arabidopsis JMJ* genes compared to *O. sativa*. Exceptions included *VvJMJ18* and *VvJMJ20*, *VvJMJ5* and *OsJMJ719* in the KDM3 subfamily, *VvJMJ7* and *VvJMJ9* in the JMJC subfamily, *VvJMJ2* and *VvJMJ19* in the KDM4 subfamily, and *VvJMJ13* and *OsJMJ704* in the KDM5 subfamily, which formed separate clusters.

**Fig. 3:**
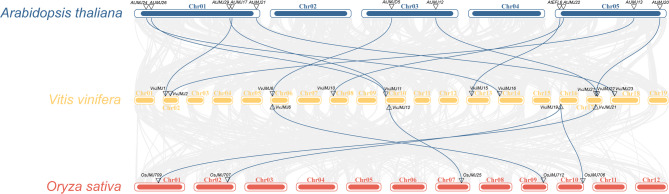
Synteny analysis of *JMJs* of *V. vinifera*, *Arabidopsis*, and *O. sativa*

We performed a comparative syntenic analysis of *V. vinifera*, *Arabidopsis*, and *O. sativa* genomes to better understand the origin, evolution, and function of *JMJ* genes (Fig. 3). As shown in Fig. 3, there were a total of 11 syntenic gene pairs between *V. vinifera* and *Arabidopsis*, which included *VvJMJ1*-*AtJMJ17*, *VvJMJ2*-*AtJMJ13*, *VvJMJ6*-*AtJMJD5*, *VvJMJ10*-*AtJMJ22*, *VvJMJ11*-*AtJMJ29*, *VvJMJ11*-*AtJMJ26*, *VvJMJ15*-*AtEFL6*, *VvJMJ16*-*AtJMJ24*, *VvJMJ21*-*AtJMJ20*, *VvJMJ22*-*AtJMJ12*, and *VvJMJ23*-*AtJMJ21*. There were five syntenic gene pairs between *V. vinifera* and *O. sativa*, namely *VvJMJ6*-*OsJMJ712*, *VvJMJ12*-*OsJMJ25*, *VvJMJ19*-*OsJMJ707*, *VvJMJ19*-*OsJMJ706*, and *VvJMJ21*-*OsJMJ709*. The syntenic gene count suggested that the *V. vinifera JMJ* genome is more conserved with *Arabidopsis* compared to its more distant relationship with *O. sativa*. The specific information for the collinear genes between species is shown in Table S3.

### Sequence and structure analysis of *JMJs*

**Fig. 4:**
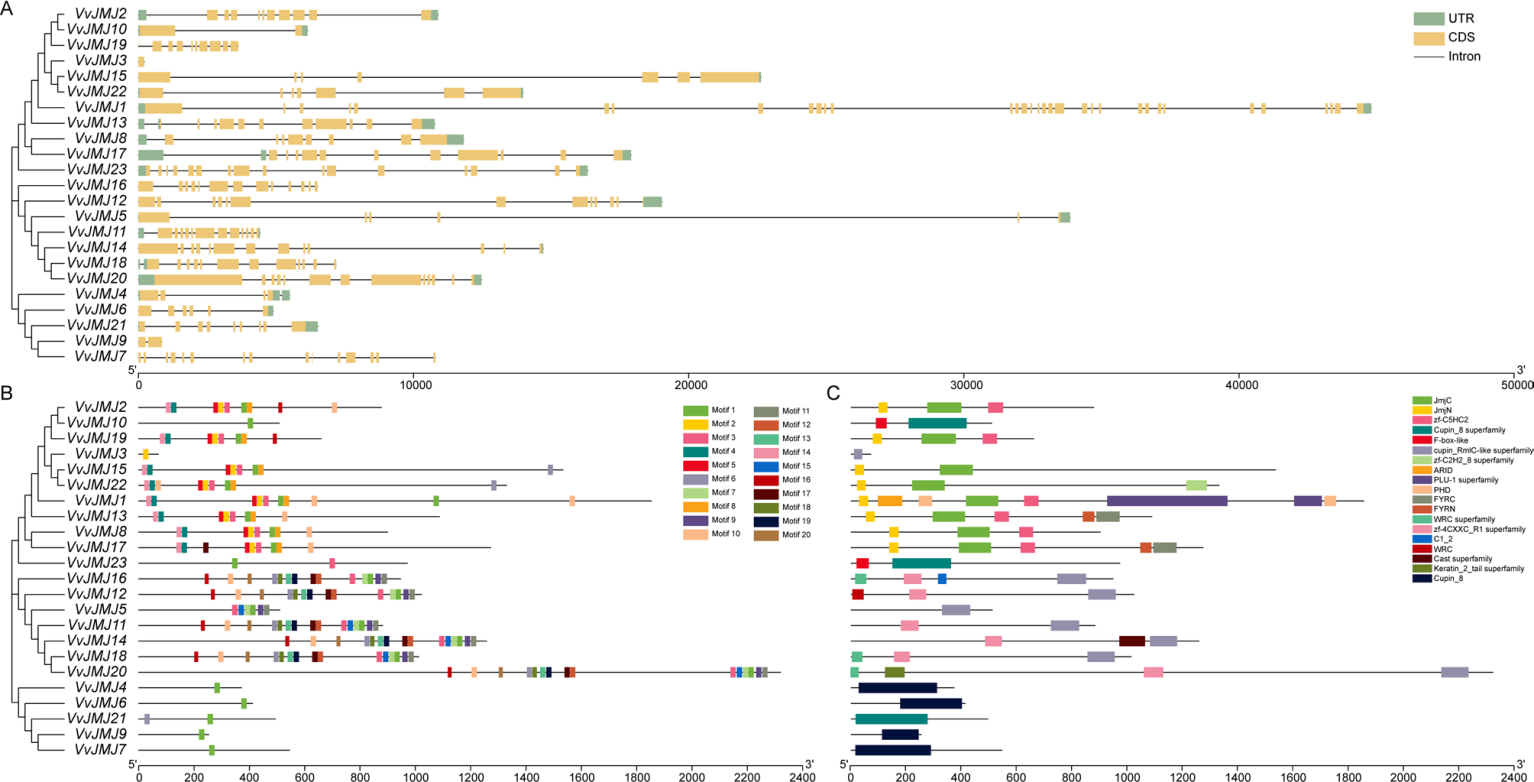
Phylogenetic analysis, motif composition, conserved domains of VvJMJ proteins and exon–intron structures of *VvJMJ* genes. The phylogenetic tree and gene structures of the *VvJMJ* genes (**A**), motif composition (**B**) and the distribution of conserved domains (**C**) of VvJMJs. Green rectangles represent untranslated regions (UTRs); yellow rectangles represent coding sequence (CDS) or exons; grey lines represent introns

The gene structure analysis showed that within the KDM4 subfamily (*VvJMJ2* ~ *VvJMJ22*), except for *VvJMJ3* which had one exon, the remaining members had 7 to 10 exons; within the KDM5 subfamily (*VvJMJ1* ~ *VvJMJ17*), *VvJMJ1* had 33 exons, *VvJMJ13* and *VvJMJ17* had 11 exons, while *VvJMJ8* had eight exons; within the JMJD6 subfamily (*VvJMJ10*, *VvJMJ23*), *VvJMJ10* had two exons, and *VvJMJ23* had 16 exons; within the KDM3 subfamily (*VvJMJ16* ~ *VvJMJ20*), except for VvJMJ5 with six exons and *VvJMJ11* and *VvJMJ18* with 12 exons, *VvJMJ16*, *VvJMJ12*, *VvJMJ14*, and *VvJMJ20* all had 13 exons; within the JMJC subfamily (*VvJMJ4* ~ *VvJMJ7*), the number of exons varies among the genes, with *VvJMJ4* having four exons, *VvJMJ6* having six exons, *VvJMJ21* having nine exons, *VvJMJ9* having two exons, and *VvJMJ7* having 15 exons (Fig. 4A).

Based on the annotation information of the *V. vinifera JMJ* genes, a motif structure diagram was constructed (Fig. 4B). The analysis showed that within the KDM4 subfamily (VvJMJ2 ~ VvJMJ22), except for VvJMJ3 which contained only one Motif 2, the remaining members all contained multiple motif sequences; within the KDM5 subfamily (VvJMJ1 ~ VvJMJ17), each member contained eight to ten motif sequences; within the JMJD6 subfamily (VvJMJ10, VvJMJ23), VvJMJ10 contained one Motif 1, and VvJMJ23 contained one Motif 1 and one Motif 3; within the KDM3 subfamily (VvJMJ16 ~ VvJMJ20), except for VvJMJ16 and VvJMJ12 lacking Motif 15, and VvJMJ5 having only 6 motifs, the remaining genes all contain 15 motifs, and the motif types and arrangements were completely consistent. In the JMJC subfamily (VvJMJ4 to VvJMJ7), all genes except *VvJMJ21*, which included Motif 1 and Motif 6, contain only Motif 1. This suggested that Motif 1 is the conserved motif within this subfamily.

Conserved domain analysis corroborated the motif analysis, reinforcing the similarity in domain structures within gene subfamilies (Fig. 4C). Conserved domain analysis of the KDM4 subfamily (VvJMJ2 to VvJMJ22) revealed that all genes, except VvJMJ3 which hasd a single cupin_RmlC-like superfamily domain, possess two conserved domains: JmjC and JmjN. In the KDM5 subfamily (VvJMJ1 to VvJMJ17), all genes feature three conserved domains: JmjC, JmjN, and the zinc finger structure zf-C5HC2. Additionally, VvJMJ13 and VvJMJ17 had two FYRC and FYRN domains, while VvJMJ1 included three domains: ARID, two copies of PHD, and two copies of the PLU-1 superfamily. The JMJD6 subfamily (VvJMJ10, VvJMJ23) contained two conserved domains: Cupin 8 superfamily and F-box-like. In the KDM3 subfamily (VvJMJ16 to VvJMJ20), all genes except VvJMJ5, which had only a cupin_RmlC-like superfamily domain, contain two domains: cupin_RmlC-like superfamily and zf-4CXXC_Rl superfamily. Within the JMJC subfamily (VvJMJ4 to VvJMJ7), all genes except VvJMJ21, which hasd a Cupin 8 superfamily domain, contain a single Cupin 8 domain. Detailed amino acid sequences of *VvJMJ* motifs and MEME site analysis were provided in Supplementary Table S4.

### Cis-elements in the promoter of *VvJMJs*

**Fig. 5:**
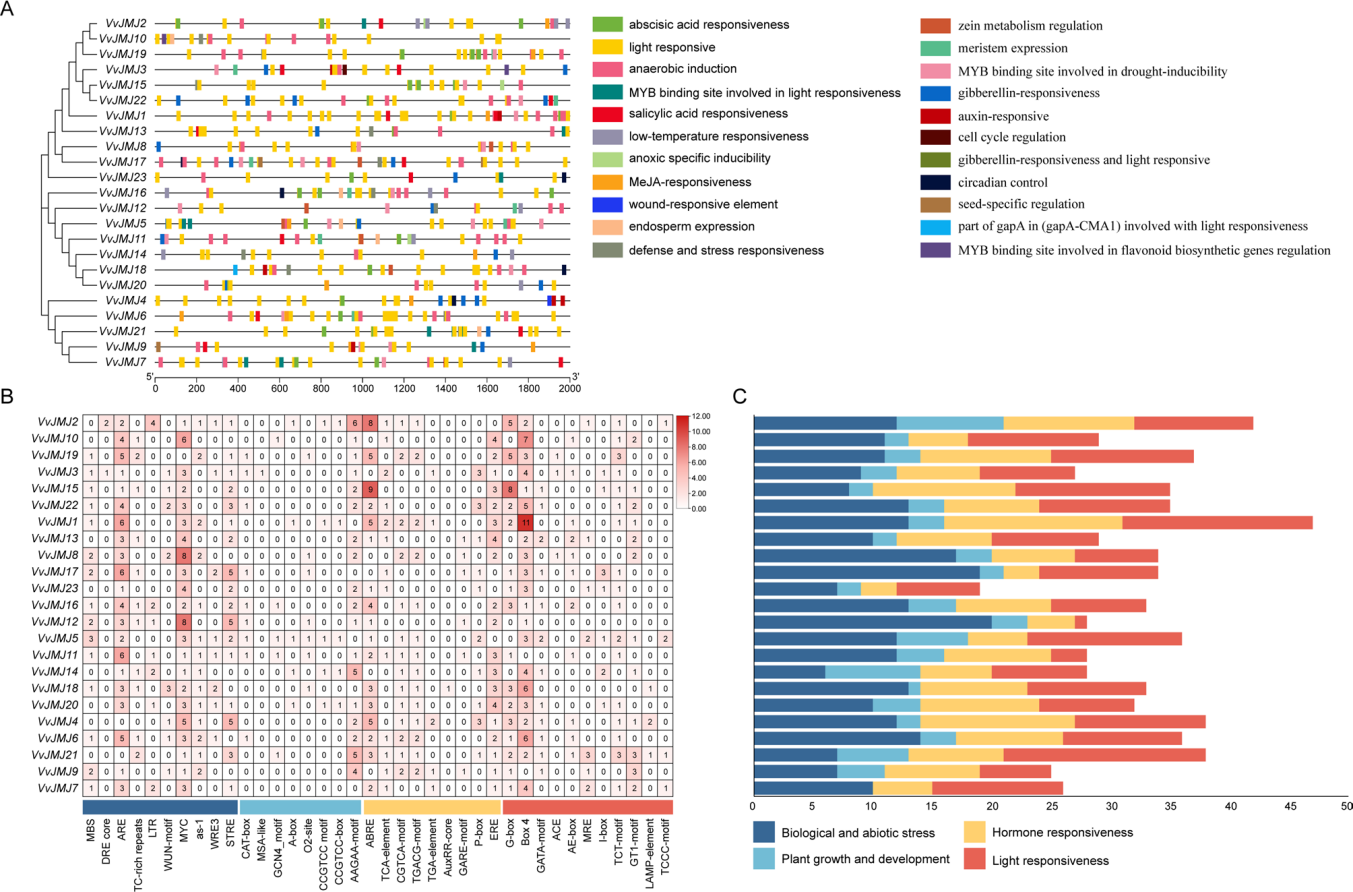
The *cis*-elements analysis of *VvJMJ* promoters. The distribution (**A**) and number (**B** and **C**) of *cis*-elements in the promoter of *VvJMJ* genes

Researchers had identified 22 *cis*-elements in the promoter sequences of *V. vinifera JMJ* family genes to explore their regulatory mechanisms and stress responses. These elements included those responsive to abscisic acid, light, anaerobic conditions, salicylic acid, low temperatures, MeJA, wounds, and gibberellin, as well as MYB binding sites related to light and drought, defense and stress response, zein metabolism, meristem and endosperm expression, cell cycle regulation, circadian control, seed-specific regulation, and a gapA element associated with light responsiveness. According to their functional roles, these elements are divided into four major categories: non-biological and biological stress responses, plant growth and development, plant hormone responses, and light responses (Fig. 5B and C). All *VvJMJs* gene promoters contained these four types of *cis*-elements.

The non-biological and biological stress response category included 10 *cis*-elements, with 266 elements in total in *VvJMJs* genes, including: 70 MYC elements, the highest percentage being 26.3%, with the exception of *VvJMJ19* which did not contain this element; 38 STRE elements, with six genes (*VvJMJ10*, *VvJMJ8*, *VvJMJ14*, *VvJMJ18*, *VvJMJ6*, and *VvJMJ9*) not containing this element; 67 ARE elements for anaerobic induction, the second highest percentage being 25.2%, with the exception of *VvJMJ4* and *VvJMJ21* which did not contain this element; 21 MBS elements for drought stress, with *VvJMJ5* containing the most, three elements; three DRE core elements, the lowest percentage being 1.1, with only *VvJMJ2* and *VvJMJ3* containing two and one elements, respectively.

The plant growth and development category comprised eight *cis*-acting elements, totaling 79 in *VvJMJs* genes. Among these, 42 were AAGAA-motif elements, constituting 53.2%, except in VvJMJ1, VvJMJ17, VvJMJ5, VvJMJ18, and VvJMJ7, which lacked this element. Other elements included nine CAT-box elements for fungal tissue expression, five each of GCN4_motif, A-box, CCGTCC motif, and CCGTCC-box elements, and seven O2-site elements. The MSA-like element was the least common, present only in *VvJMJ3*.

The plant hormone response category included nine *cis*-elements, with 184 elements in total in *VvJMJs* genes, including: 57 ABRE elements for abscisic acid responsiveness, the highest percentage being 30.5%, with *VvJMJ15* and *VvJMJ2* containing nine and eight ABRE elements, respectively; 44 ERE elements for ethylene responsiveness, the second highest percentage being 23%; four *VvJMJs* genes (*VvJMJ10*, *VvJMJ13*, and *VvJMJ20*) containing the most ERE elements, four each; The CGTCA-motif and TGACG-motif elements, accounting for 10.2%, were associated with MeJA responsiveness. 17 P-box and six GARE-motif elements for gibberellin responsiveness; 15 TCA-element elements for salicylic acid responsiveness; six TGA-element and one AuxRE-core element for auxin responsiveness, with only *VvJMJ18* containing one AuxRE-core element.

The light response category included 11 *cis*-elements, with 220 elements in total in *VvJMJs* genes, including: 76 Box four elements for a part of the conserved DNA light response, the highest percentage being 34.5%, with *VvJMJ1*, *VvJMJ10*, *VvJMJ6*, and *VvJMJ18* containing 11, seven, six, and six elements, respectively; 41 G-box elements for light-responsive *cis*-regulatory elements, the second highest percentage being 18.6%, with *VvJMJ15*, *VvJMJ19*, and *VvJMJ2* containing eight, five, and five elements, respectively; four LAMP-element elements, the lowest percentage being 1.8%, with *VvJMJ4*, *VvJMJ21*, and *VvJMJ18* containing two, one, and one element, respectively; three ACE elements, the lowest percentage being 1.4%, with *VvJMJ19*, *VvJMJ3*, and *VvJMJ8* each containing one element.

In the *V. vinifera JMJ* gene family, *VvJMJs* genes contained a significant number of MYC elements, AAGAA-motif elements, abscisic acid response elements ABRE, and conservative DNA light response elements Box 4. These results suggested that they are important response elements for inducing *VvJMJs* gene expression.

### Expression profile of *VvJMJ* in the development process of fruits

**Fig. 6:**
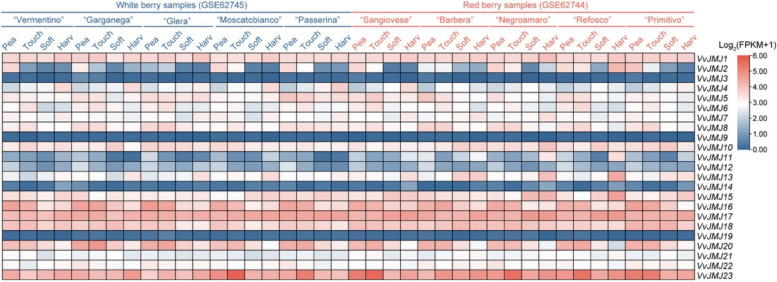
Expression profiles of *V. vinifera VvJMJ* genes in different varieties and different developmental stages of fruits. Different shades of red and blue denote the extent of the expression values according to the color bar provided [log_2_(FPKM + 1)]

This study analyzed *VvJMJs* expression profiles during *V. vinifera* fruit development across various varieties using publicly available transcriptome data (Fig. 6). The findings indicated that *VvJMJ3*, *VvJMJ9*, *VvJMJ14*, and *VvJMJ19* had reduced expression levels across both the five white and five red fruit varieties. *VvJMJ14* exhibited marginally elevated expression levels during the Pea and Harv developmental stages in the five red fruit varieties compared to the other stages. *VvJMJ16* and *VvJMJ20* exhibited elevated expression levels during the Pea and Touch developmental stages across ten *V. vinifera* varieties, compared to the Soft and Harv stages. Notably, *VvJMJ20* expression gradually declined as the fruit matured, indicating a potential role for these genes in *V. vinifera* fruit development. The expression profile of *VvJMJ23* varied among different varieties, exhibiting elevated levels during the Touch developmental stage in the white fruit varieties'Moscatobianco'and'Passerina'. *VvJMJ23* exhibited elevated expression levels during various developmental stages in the fruits of four red fruit varieties:'Sangiovese'(Pea and Touch),'Negroamaro'(Touch),'Refosco'(Touch), and'Primitivo'(Pea and Touch). The expression trend of *VvJMJ2* is notable, as its overall expression levels decrease during the middle and late stages of fruit development across all varieties, except for'Refosco'. *VvJMJ4* exhibited elevated expression levels during the Harv developmental stage in the five white fruit varieties, whereas in the red fruit varieties, expression was typically lower, with the exception of the'Sangiovese'and'Primitivo'varieties. The results suggested that certain *VvJMJ* genes may be involved in *V. vinifera* fruit development and exhibit varietal differences.

*V. vinifera* fruit samples (*V. vinifera* L. cv. Shine Muscat) were collected from the same location and under the same cultivation conditions at four different developmental stages of the fruit in the same year. The four stages were 20 days after flowering (Pea), before the fruit color change (Touch), at the end of the fruit color change (Soft), and at the time of full maturity and harvest (Harv). Samples from each stage were collected in triplicate and promptly preserved in liquid nitrogen for total RNA extraction.

**Fig. 7:**
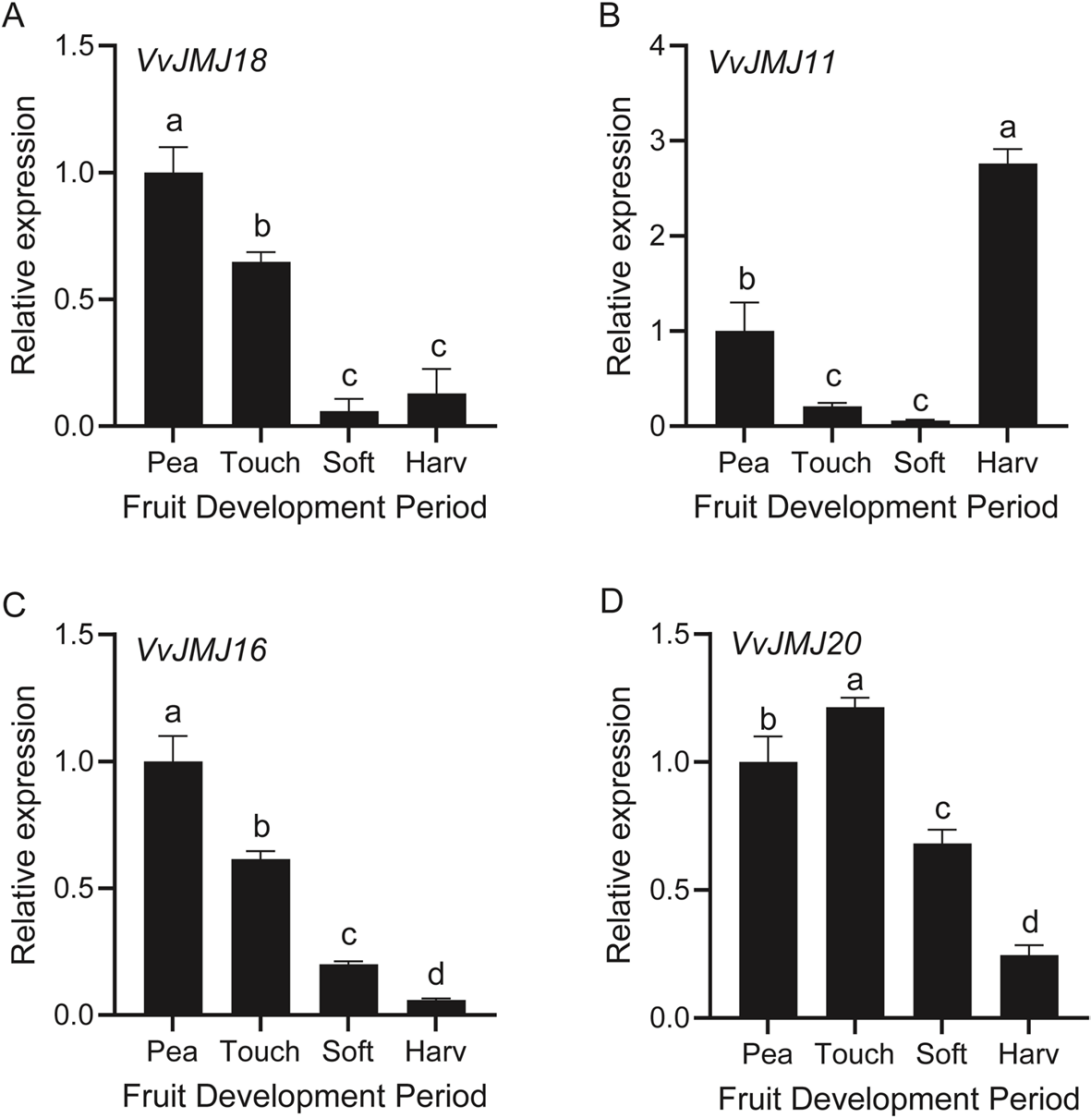
The qRT-PCR analysis of some *VvJMJ* genes during the development of *V. vinifera* fruits. The mean values ± SEM are shown for three biological replicates. Different letters above the bars indicate significant differences according to Duncan-test (*p* < 0.05)

To confirm the expression trends of *VvJMJ* genes during *V. vinifera* fruit development, four *VvJMJ* genes were analyzed using qRT-PCR (Fig. 7). The analysis indicated that *VvJMJ18* expression progressively declined during fruit maturation, reaching a minimum at the Soft stage. *VvJMJ11* expression decreased slowly across the initial three developmental stages, followed by a sharp increase at the Harv stage, suggesting its role in the final maturation phase. *VvJMJ16* expression consistently decreased as the *V. vinifera* fruit developed, while *VvJMJ20* expression peaked at the Touch stage before declining. The above results were basically consistent with the transcriptome analysis results.

### Tissue-specific expression patterns of *VvJMJ* genes in *V. vinifera*

**Fig. 8:**
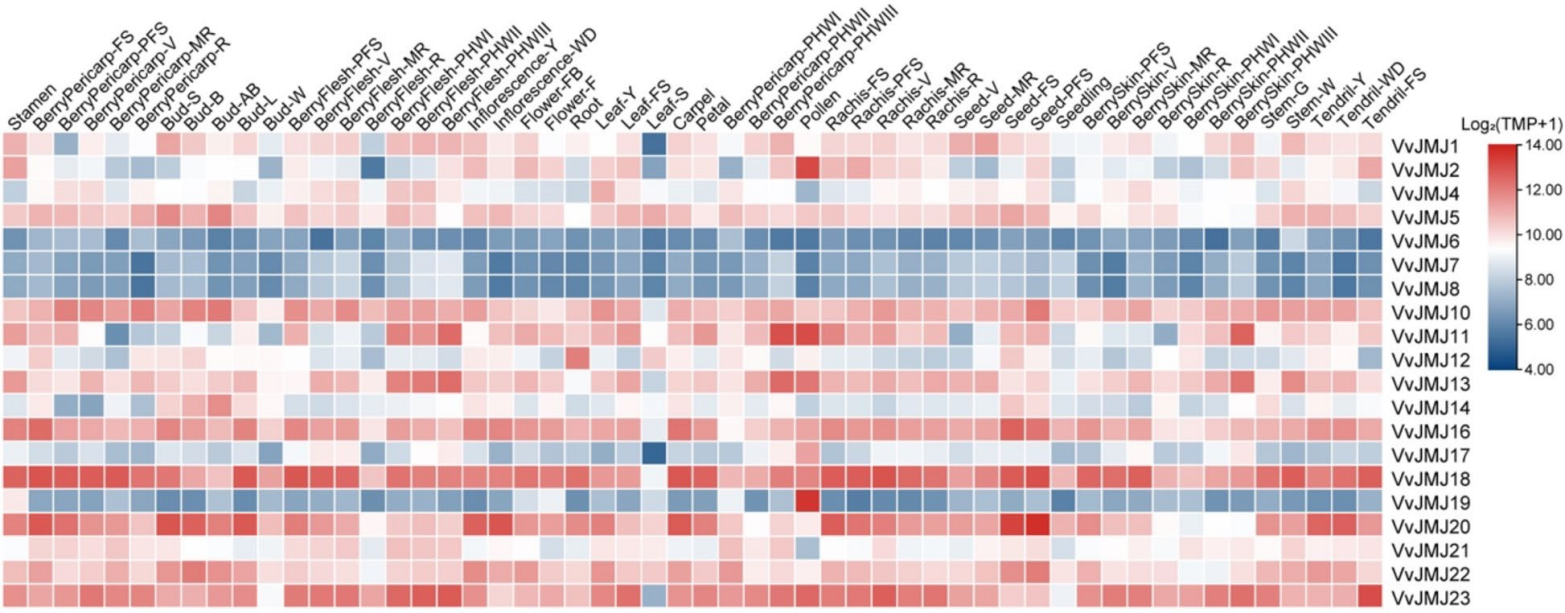
Expression profiles of grapevine *VvJMJ* genes in various tissues and developmental stages. Different shades of red and blue denote the extent of the expression values according to the color bar provided [log2(FPKM + 1)]. Bud-AB, bud after burst; Bud B, Bud burst; Bud-W, winter bud; Bud-L, latent bud; Bud-S, bud swell; Flower-F, flowering; Flower-FB, flowering begins; FS, fruit set; Inflorescence-Y, young inflorescence with single flowers separated; Inflorescence-WD, well-developed inflorescence; Leaf-FS, mature leaf; Leaf-S, senescing leaf; Leaf-Y, young leaf; MR, mid-ripening; R, ripening; PFS, post fruit set; Stem-G, green stem; Stem-W, woody stem; V, véraison

To gain more insights in potential roles of *VvJMJ* genes during grapevine development, the organic specific expression patterns of all the *VvTCP* genes were analyzed using an expression atlas of *V. vinifera* cv. ‘Corvina’ from the GEO DataSets (GSE36128), which contained 42 various organs/tissues at different developmental stages obtained by microarray analysis. As shown in Fig. 8, some *VvJMJ* genes exhibited similar expression patterns across different tissues, while others displayed significant tissue specificity, suggesting functional differentiation of *VvJMJ* genes during the development of various *V. vinifera* organs or tissues. For instance, *VvJMJ18* showd relatively high expression levels in all tested tissues, whereas *VvJMJ6*, *VvJMJ7*, and *VvJMJ8* demonstrated low expression across tissues. Notably, *VvJMJ2* and *VvJMJ19* exhibited minimal expression in most tissues but show significantly elevated expression in pollen, indicating their potential involvement in *V. vinifera* pollen formation. Similarly, *VvJMJ11* and *VvJMJ13* both display marked tissue-specific high expression in pollen, suggesting their association with pollen development and fertility. *VvJMJ5* showed elevated expression during fruit skin development and bud swelling phases, potentially regulating fruit skin maturation and playing critical roles in bud expansion. *VvJMJ10* maintained sustained high expression during postharvest fruit ripening, likely influencing post-storage fruit quality. These findings provided important clues for further investigation into the transcriptional regulatory mechanisms of *VvJMJ* genes during *V. vinifera* fruit development and ripening processes.

### Expression profiles of the *Vitis vinifera VvJMJs* in response to hormone treatment

**Fig. 9:**
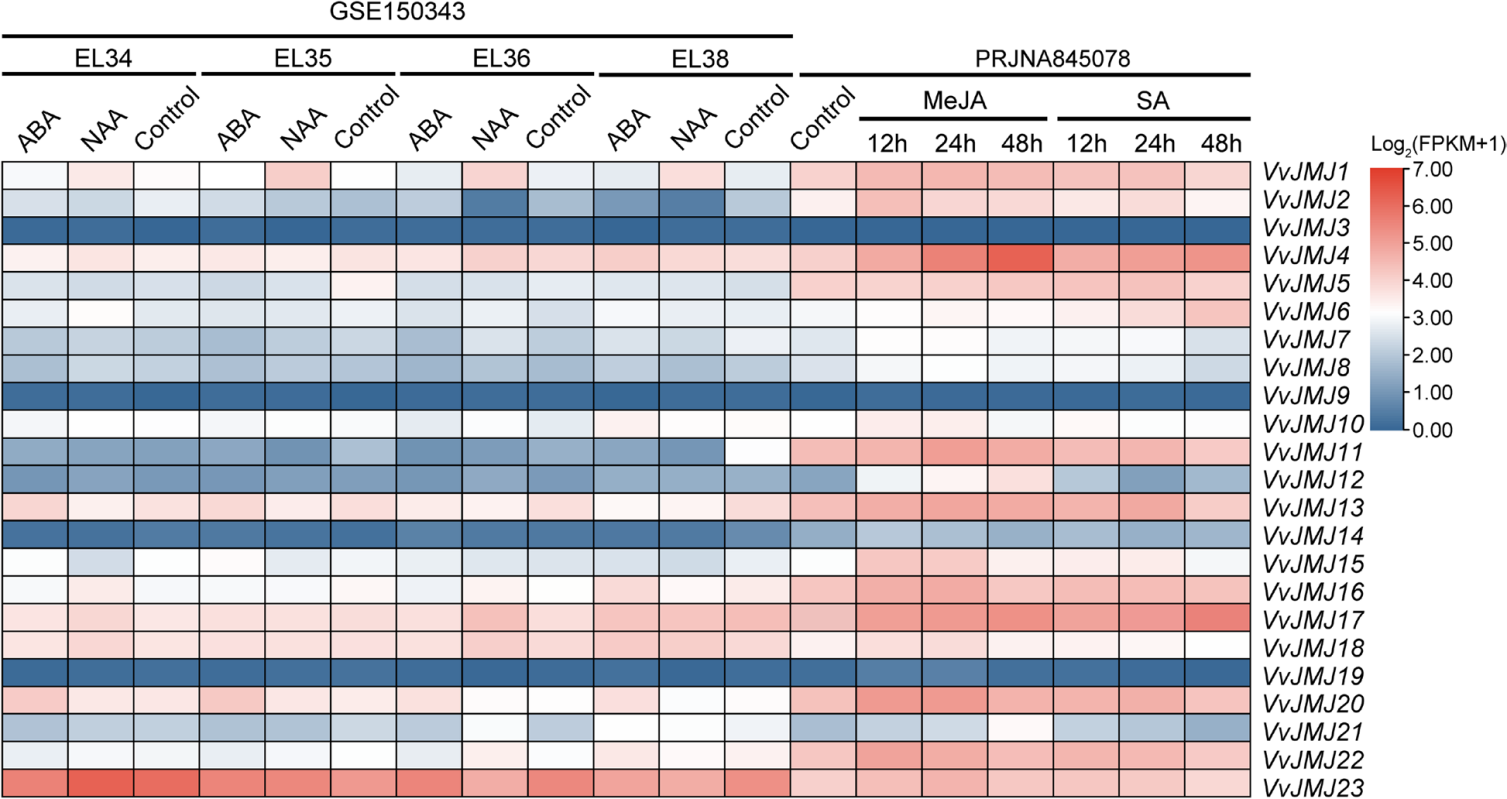
Expression profiles of *VvJMJ* genes in fruits and leaves under corresponding hormone treatments in *V. vinifera*. Different shades of red and blue denote the extent of the expression values according to the color bar provided [log2(FPKM + 1)]

Plant hormones are essential for plant growth and development [42]. We examined *VvJMJs* gene expression profiles in response to hormone treatments with NAA, ABA, MeJA, and SA using publicly accessible transcriptome data (Fig. 9). The findings indicated that *VvJMJ3*, *VvJMJ9*, and *VvJMJ19* had minimal or no expression across all four hormone treatments, while *VvJMJ14* demonstrated reduced expression specifically under ABA and NAA treatments. In *V. vinifera* fruits treated with NAA, *VvJMJ1* expression significantly increased at stages EL34, EL35, EL36, and EL38 compared to the control, whereas *VvJMJ13* expression slightly decreased. ABA treatment in *V. vinifera* fruits resulted in a significant increase in the expression levels of *VvJMJ20* compared to the control. In *V. vinifera* leaves treated with MeJA, the expression levels of *VvJMJ4*, *VvJMJ12*, and *VvJMJ21* consistently increased over time, whereas *VvJMJ2*, *VvJMJ10*, *VvJMJ20*, and *VvJMJ22* initially increased and then decreased. Under SA treatment, the expression levels of *VvJMJ4*, *VvJMJ6*, and *VvJMJ17* progressively rose with prolonged treatment, whereas *VvJMJ21* exhibited a slight decline. In summary, we speculated that the above genes may participate in the process of *V. vinifera* response to different hormone treatments and play an important role.

We selected some *VvJMJ* genes for qRT-PCR analysis after MeJA and SA treatments. One-year-old grapevines (*V. vinifera* L. cv. Shine Muscat) grapes were grown in a light incubator set to 25 °C with 65% relative humidity, following a 16 h light and 8 h dark cycle. Plants with similar growth conditions were selected as experimental materials. MeJA (Sigma Aldrich Chemicals GmbH, Schnelldorf, Germany) was prepared at a final concentration of 100 μM by dissolving it in 10% ethanol with 0.2% Tween-20, using 0.2% Tween-20 as the control. Before spraying, the leaves were washed with distilled water and then dried before being evenly sprayed onto the surface of the grapevine leaves using a spray bottle. Leaves were collected at 12, 24, and 48 h post-spraying for total RNA extraction, with three leaves sampled at each interval and preserved in liquid nitrogen. SA (Sigma Aldrich Chemicals GmbH, Schnelldorf, Germany) was prepared at a concentration of 100 μM in 10% ethanol, with distilled water serving as the control. The spraying method and sampling method were the same as those for the MeJA treatment.

**Fig. 10:**
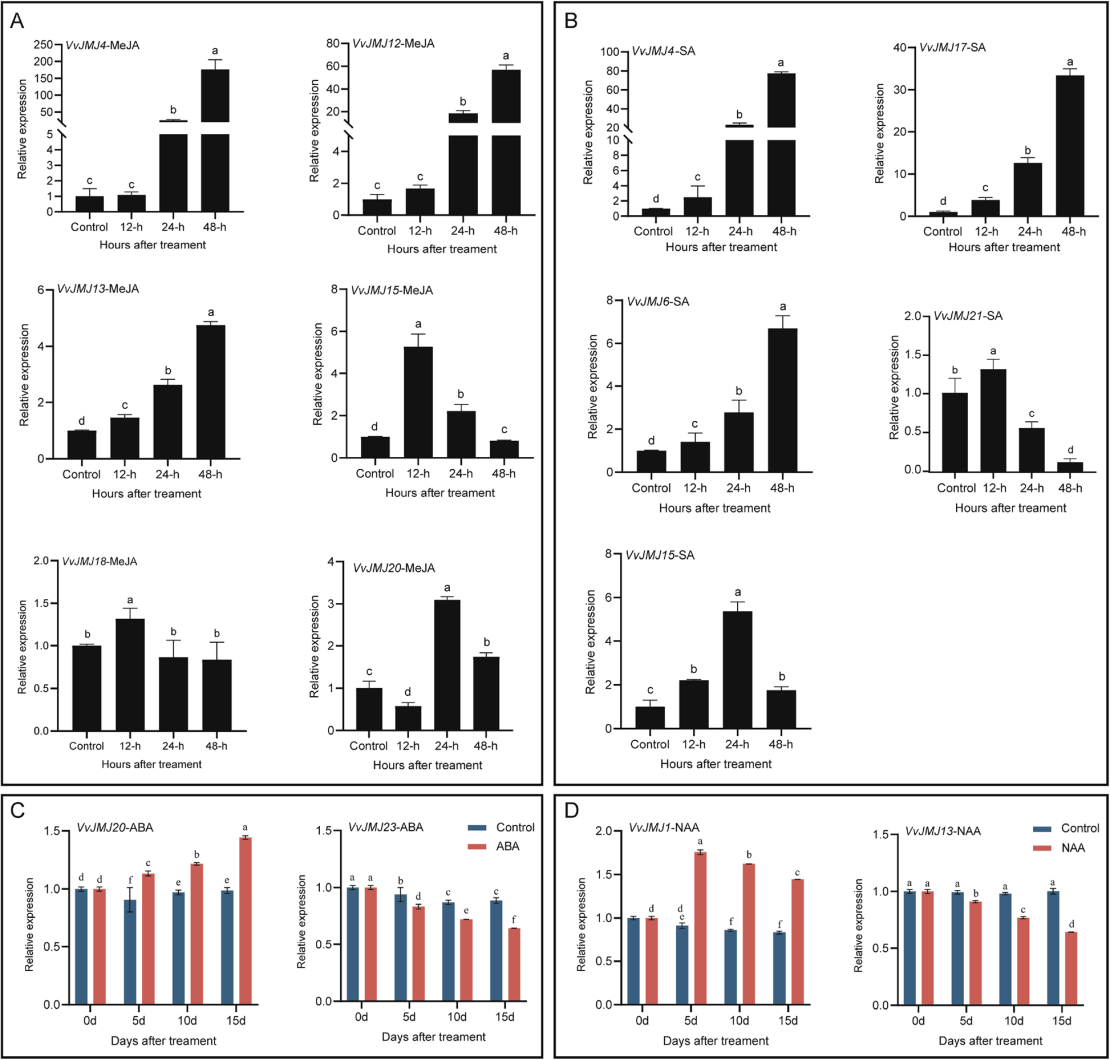
The qRT-PCR analysis of some *VvJMJ* genes under MeJA, SA, NAA and ABA treatments. The leaves of annual *V. vinifera* were sprayed with MeJA and SA, with 0.2% Tween-20 and distilled water serving as controls, and samples were collected 12 h, 24 h, and 48 h post-spraying, with three biological replicates. Using six-year-old *V. vinifera* cv.'Muscat Hamburg'as experimental material, the fruits were sprayed with ABA and NAA solutions, while a control group was treated with distilled water containing 0.05% Tween-20. Sampling was conducted at 0, 5, 10, and 15 d post-treatment. Each treatment included three biological replicates to ensure statistical reliability. The mean values ± SEM are shown for three biological replicates. Different letters above the bars indicate significant differences according to Duncan-test (*p* < 0.05)

The study found that MeJA treatment significantly upregulated the expression of *VvJMJ4*, *VvJMJ12*, and *VvJMJ13*. *VvJMJ15* and *VvJMJ18* showed increased expression at 12 h post-treatment before declining, while *VvJMJ20* exhibited a pattern of initial decrease, followed by an increase, and then a subsequent decrease (Fig. 10A). Following SA treatment, *VvJMJ4*, *VvJMJ16*, and *VvJMJ17* showed significant upregulation, while *VvJMJ21* peaked at 12 h and *VvJMJ15* at 24 h post-treatment, both subsequently declining (Fig. 10B). The above results were basically consistent with the transcriptome analysis results.

**Fig. 11:**
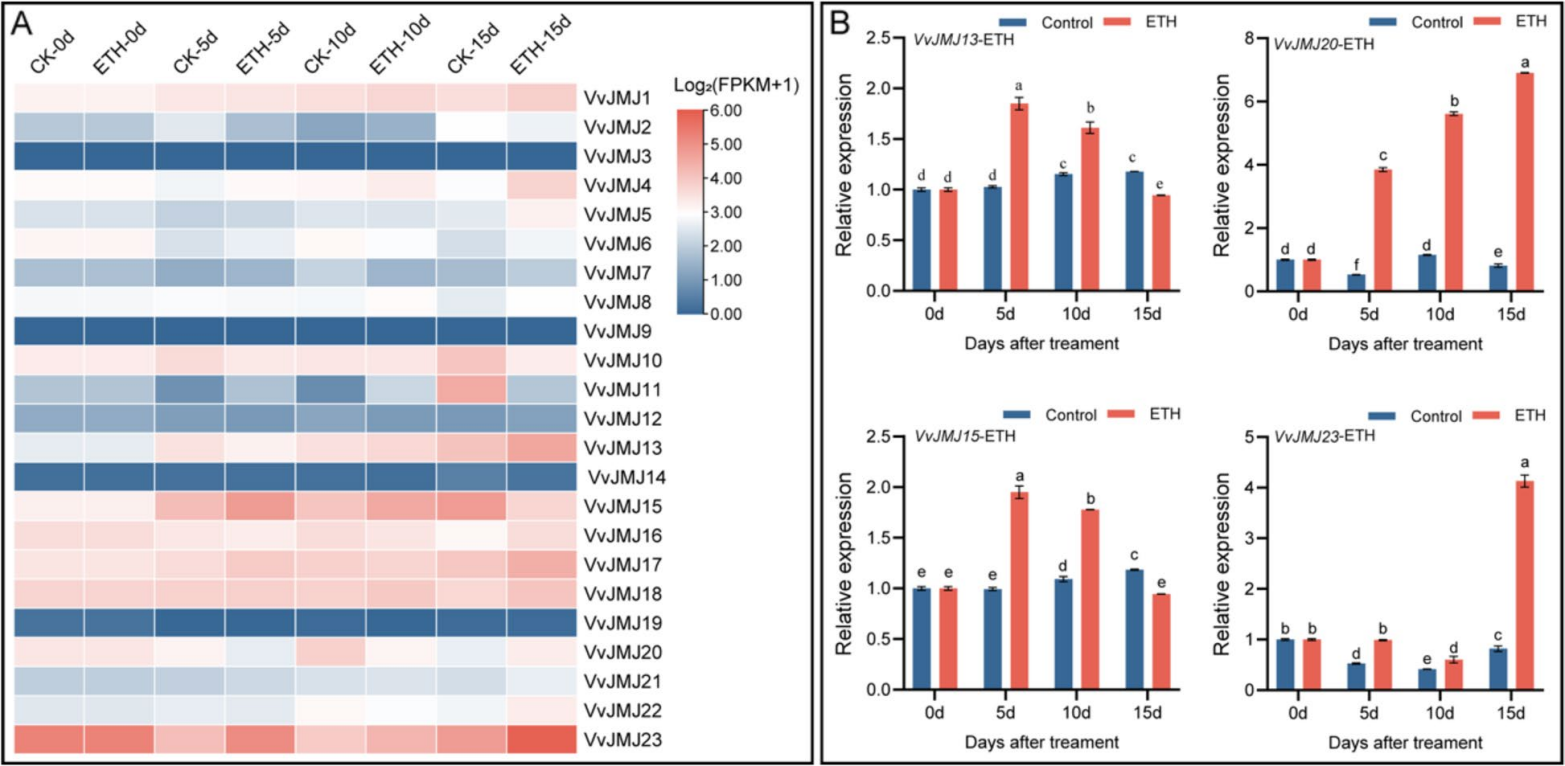
Expression profiles of *VvJMJ* genes in *V. vinifera* fruits under ETH treatment and qRT-PCR analysis of selected *VvJMJ* genes. Different shades of red and blue denote the extent of the expression values according to the color bar provided [log2(FPKM + 1)]. The mean values ± SEM are shown for three biological replicates. Different letters above the bars indicate significant differences according to Duncan-test (*p* < 0.05)

To further clarify the role of the *VvJMJ* gene family in the response of *V. vinifera* fruits to ethylene (ETH), we conducted RNA-seq analysis on fruits treated with ETH. The analysis results were shown in Fig. 11, where *VvJMJ3*, *VvJMJ9*, *VvJMJ14*, and *VvJMJ19* were found to be in a state of very low or no expression. The expression level of *VvJMJ15* on the 5 d and 10 d after ETH treatment was significantly higher than that of the control group. Notably, the expression levels of *VvJMJ23* at all three time points (5d, 10 d, and 15 d) after ETH treatment were higher than those of the corresponding control group. Furthermore, we performed qRT-PCR analysis on selected *VvJMJ* genes. The results revealed that the relative expression levels of *VvJMJ13* and *VvJMJ15* were significantly higher in ETH-treated fruits compared to the control group at 5 and 10 d post-treatment. In contrast, the relative expression of *VvJMJ20* increased progressively with treatment duration, showing significant elevation at 5, 10, and 15 d compared to the control. Notably, *VvJMJ23* exhibited a decline in expression at 10 d but a marked increase at 15d. These findings suggested that these genes may play roles in mediating *V. vinifera* fruit responses to ethylene signaling.

### Expression profiles of *VvJMJs* in grapevine under biotic and abiotic stresses

**Fig. 12:**
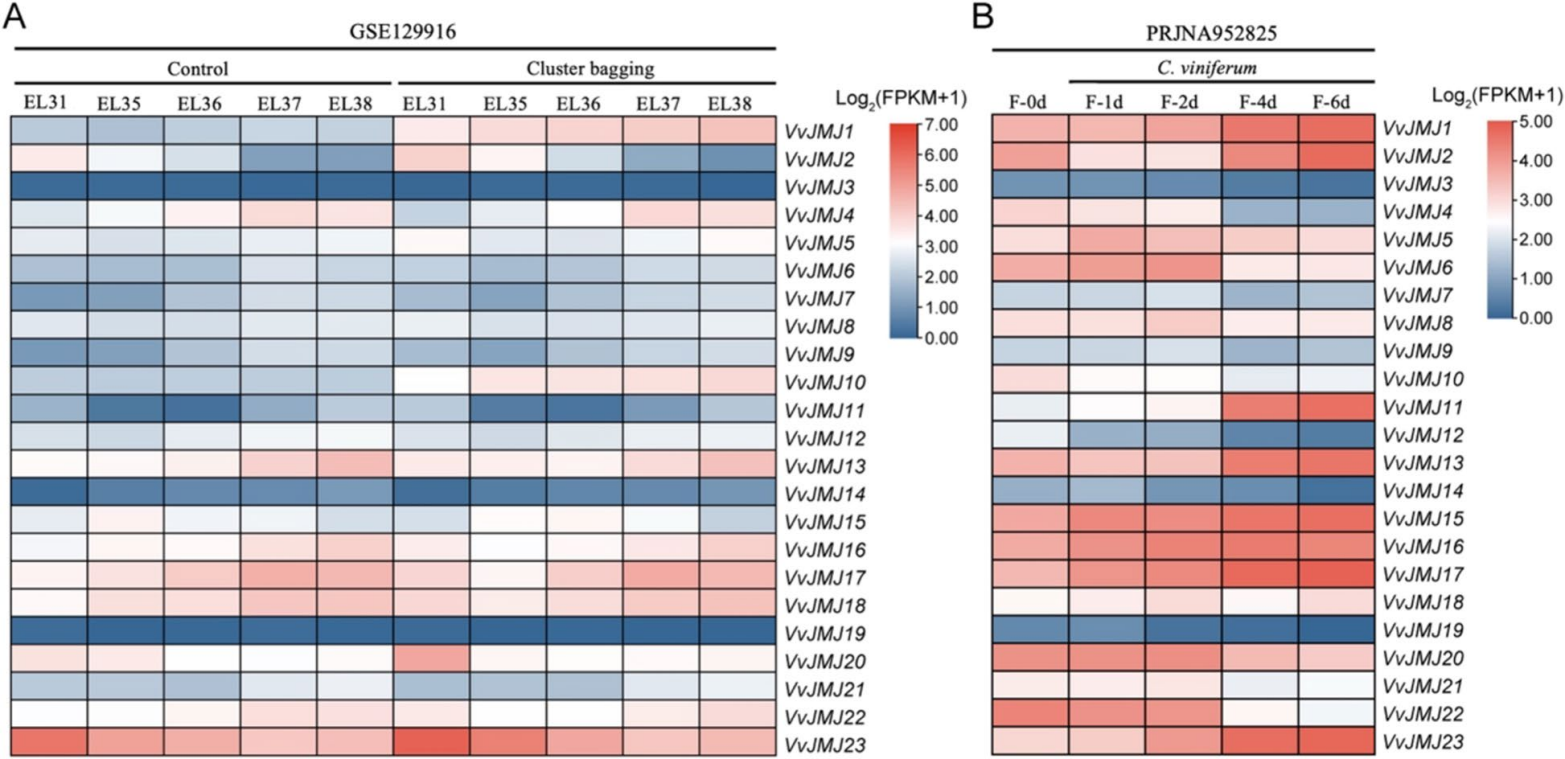
Transcriptional profiles of *VvJMJs* genes in *V. vinifera* under photostress and biotic stress responses. Different shades of red and blue denote the extent of the expression values according to the color bar provided [log2(FPKM + 1)]

Light conditions are known to significantly impact *V. vinifera* berry ripening and phenolic compound accumulation [43]. We examined *VvJMJs* expression in *V. vinifera* berries at different developmental stages in response to light using publicly available transcriptome data (Fig. 12A). The findings indicated that *VvJMJ2* and *VvJMJ19* were either minimally expressed or not expressed in both the experimental and control groups. Following cluster bagging treatment, *VvJMJ1* and *VvJMJ10* exhibited elevated expression levels compared to the control group across the EL31, EL35, EL36, EL37, and EL38 stages. Their expression progressively increased with *V. vinifera* berry maturation, indicating a potential role in the berries'response to light.

*C. viniferum*, responsible for *V. vinifera* ripe rot and leaf spot, poses a significant threat to *V. vinifera* production and quality [44]. We examined the expression levels of *VvJMJ* genes in *V. vinifera* leaves subjected to *C. viniferum* infection stress using publicly available transcriptome data (Fig. 12B). The study found that the expression levels of *VvJMJ1*, *VvJMJ11*, *VvJMJ17*, and *VvJMJ23* progressively rose following infection onset, whereas *VvJMJ2* and *VvJMJ13* initially decreased before increasing. Following infection onset, the expression levels of *VvJMJ4*, *VvJMJ12*, *VvJMJ20*, and *VvJMJ22* decreased, while *VvJMJ6* initially increased before declining.

## Discussion

Chromosomal localization analysis revealed that the 23 *VvJMJ* genes are unevenly distributed among the *V. vinifera* 12 chromosomes (Fig. 1A). To better understand the systematics of the *V. vinifera VvJMJ* gene family, we constructed a phylogenetic tree with *A. thaliana* and *O. sativa* (Fig. 2). The *V. vinifera* VvJMJ family aligns with the classification of the AtJMJ family in *A. thaliana* and the *OsJMJ* family in *O. sativa*, encompassing KDM5/JARID1, KDM4/JHDM3, KDM3/JHDM2, JMJD6, and the JmjC domain. In the VvJMJ subfamily, there were seven gene members in the KDM 3 subfamily, which was the largest proportion, consistent with the research in species such as *G. hirsutum* [10] and *Betula platyphylla* [45]; in the JMJD6 subfamily, there were two gene members, which was the smallest proportion, consistent with the research in species such as *A. thaliana*, *O. sativa*, *S. lycopersicum*,* M.. nana*, and *Cucumis melo* [46]. The similar proportion of gene members across subfamilies in various species suggested conservation of these genes among different species.

Gene family expansion primarily results from tandem gene, segmental, and whole-genome duplications [47]. This study identified only one pair of duplicated genes in *V. vinifera*. In other words, the *VvJMJ* genes were more conserved during the evolutionary process of *V. vinifera*. Furthermore, using *A. thaliana* as an outgroup, we identified the types of gene duplication within the *VvJMJ* gene family using DupGen_finder. The results revealed that within the *VvJMJ* family: two gene pairs (*VvJMJ23*-*VvJMJ21* and *VvJMJ10*-*VvJMJ21*) were classified as DSD (Dispersed Duplication); eight gene pairs (*VvJMJ3*-*VvJMJ22*, *VvJMJ5*-*VvJMJ11*, *VvJMJ7*-*VvJMJ6*, *VvJMJ8*-*VvJMJ1*, *VvJMJ9*-*VvJMJ6*, *VvJMJ12*-*VvJMJ18*, *VvJMJ14*-*VvJMJ11*, and *VvJMJ19*-*VvJMJ2*) belonged to TRD (Tandem Replicated Duplication); one gene pair (*VvJMJ18*-*VvJMJ20*) was categorized as WGD (Whole Genome Duplication) (Table S5). These findings indicated that the primary mode of expansion in the *VvJMJ* gene family is transposed duplication.

Research indicates that proteins in the KDM5/JARID subfamily demethylate H3K4me1/2/3 [48], while those in the KDM4/JHDM3 subfamily target H3K9me2/3 and H3K36me2/3 [49]. The KDM3/JHDM2 subfamily is responsible for demethylating H3K9me1 and H3K9me2 [50]. Additionally, JMJD6 subfamily proteins act on H3K27me2/3 [51], and proteins with the JmjC domain remove methylation from H3K27me3 [52]. The *AtJMJ16* gene in *A. thaliana*, part of the KDM5 subfamily, suppresses leaf senescence by reducing H3K4me3 levels and inhibiting *WRKY53* and *SAG201* gene expression [15]. This suggests that the Vitis vinifera *VvJMJ17* gene, closely related to *AtJMJ16*, might play a crucial role in *V. vinifera* leaf senescence regulation. Additionally, the *AtJMJ27* gene in *Arabidopsis*, homologous to *V. vinifera VvJMJ18* and *VvJMJ20* genes, can delay flowering by downregulating *CONSTANS* (*CO*) and upregulating *FLOWSYS LOCUS C* (*FLC*) [53]. *AtJMJ27* also binds to drought stress regulators *GOLS2* and *RD20*, contributing to drought response [54]. The *AtJMJ25* gene in *A. thaliana*, closely related to the *VvJMJ14* gene within the KDM3 subfamily, is involved in early embryonic development and embryo sac formation in *A. thaliana* [55], suggesting that *VvJMJ14* may perform similar functions. The *A. thaliana* genes *AtJMJ30* and *AtJMJ32*, closely related to *VvJMJ6* and *VvJMJ4* in the JMJC subfamily, can demethylate H3K27me2/3 from FLC to inhibit early flowering under appropriate temperatures [56]. They also activate *SnRK2.8* expression by removing H3K27me3, thereby participating in the ABA response during seedling root development [57].

Gene structure analysis revealed variability in exon numbers across different subfamilies and within members of the same subfamily. However, most genes within the same subfamily, excluding the JMJC subfamily, exhibited similar exon structures (Fig. 4A). Motif structures can reflect the evolutionary situation of *JMJ* genes, and in this study, there were conserved motifs of different numbers and types in each subfamily (Fig. 4B). Protein domains are the basic structure and functional units of the tertiary structure of proteins [58]. The analysis showed that the types and numbers of structural domains between subfamilies of *V. vinifera JMJ* genes were not similar, but each subfamily had the same conserved structural domains (Fig. 4C). VvJMJ proteins contain conserved structural domains like FYRN, FYRC, Zf-C5HC2, ARID, PLU-1, and zf-4CXXC_R1, which facilitate DNA-binding and contribute to the specific functions of JmjC proteins [59]. In addition, the conserved structural domains JmjC and JmjN in the KDM4 subfamily, which are shorter in amino acid sequence length than JmjC, have been shown to disrupt the stability and activity of JmjC if removed [60]. The PHD domain of the KDM5 subfamily can identify both unmodified and methylated histone tails [61], while the PLU-1 domain is potentially involved in DNA binding and transcription [62]. The F-box domain in the JMJD6 subfamily can recognize multiple substrates and regulate important biological processes in plants by degrading cellular proteins [63].

Transcriptional regulatory elements in gene promoter regions are essential for transcription and gene expression regulation [64]. This study examined the promoter region of *V. vinifera VvJMJ* genes, identifying numerous *cis*-acting elements associated with plant hormones, abiotic stress, growth and development, and light response (Fig. 5). Key *cis*-acting elements identified include MYC, AAGAA-motif, ABRE, and Box 4. Collinear gene pairs *VvJMJ18* and *VvJMJ20* share similar *cis*-acting elements, including ARE, ABRE, G-box, and Box 4, indicating potential co-induction under non-abiotic stress, hormone, and light conditions.

Usually, the interaction between hormones affects the development and maturation of fruits [64], and abscisic acid is closely related to fruit ripening [65–67]. Studies have shown that exogenous ABA treatment promotes the softening of *Fragaria ananassa* [68] and mangoes [69], thereby affecting fruit ripening. The study identified numerous plant hormone response elements, notably the prevalent ABA response element ABRE, alongside other elements such as the ethylene response element ERE, MeJA response elements CGTCA-motif and TGACG-motif, GA3 response elements P-box and GARE-motif, SA response element TCA-element, and IAA response elements TGA-element and AuxRE-core. Transcriptional profiling revealed that *VvJMJ20* was highly expressed across all 10 V*. vinifera* varieties prior to full ripening and showed increased expression in *V. vinifera* fruits treated with ABA compared to the control. The promoter region of *VvJMJ20* contains three ABA response elements (ABRE), indicating its role in regulating the ABA response during *V. vinifera* fruit ripening. MeJA, a plant hormone and signaling molecule associated with damage, is commonly found in plants. Its exogenous application can induce the expression of defense-related genes [70]. Both transcriptional profiling and qRT-PCR analysis revealed that *VvJMJ4* and *VvJMJ12* gene expression significantly increased during exogenous MeJA treatment. Conversely, their expression levels significantly decreased following infection in the transcriptional profiling analysis of biotic stress. This suggests that *VvJMJ4* and *VvJMJ12* may be involved in the biotic stress response mediated by MeJA signaling.

**Fig. 13:**
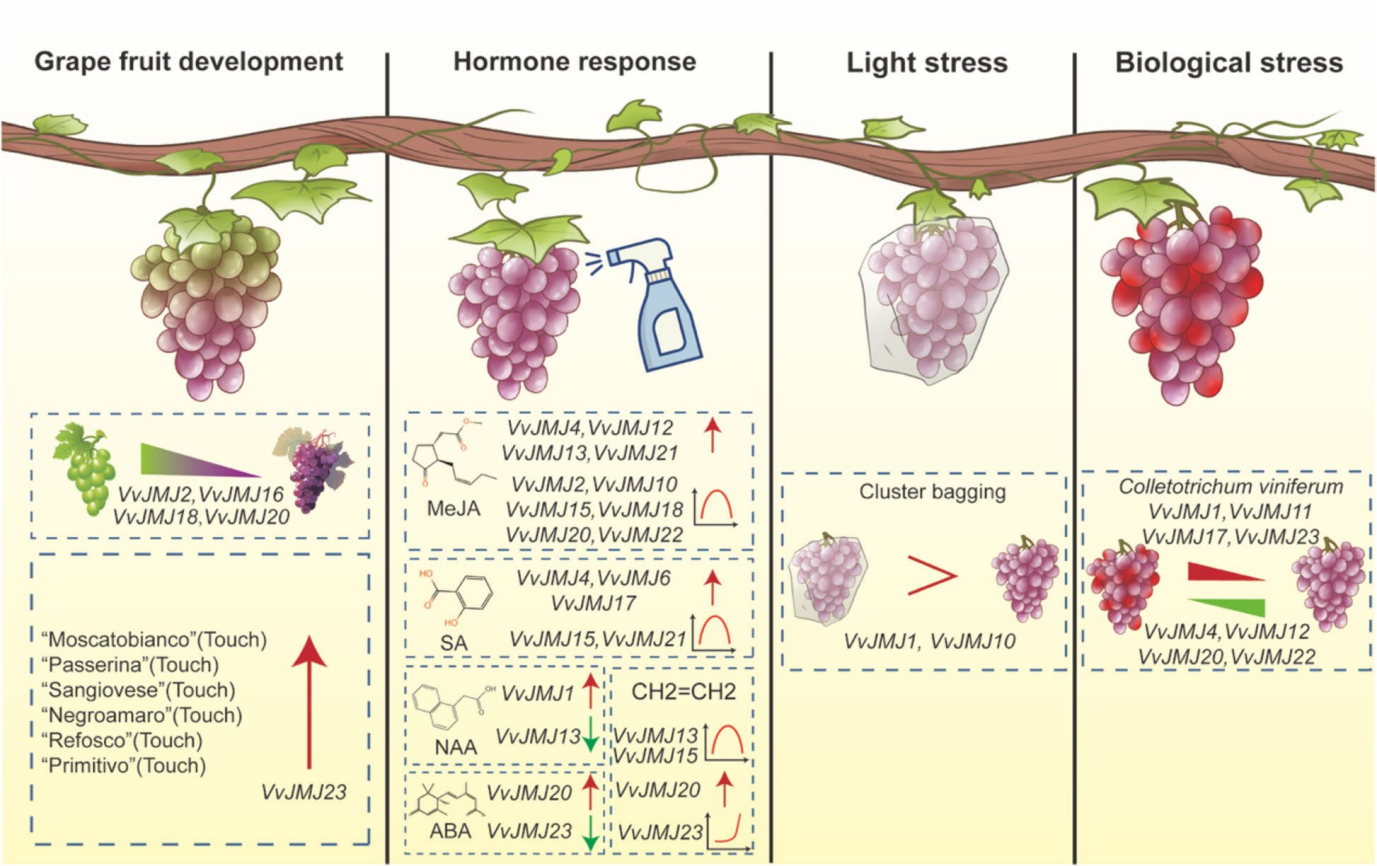
The role the *VvJMJ* genes in *V. vinifera*

This study examined *VvJMJ* gene expression patterns throughout *V. vinifera* fruit development and their reactions to biotic and abiotic stresses, as well as hormone treatments. Analysis of *VvJMJ* gene expression profiles suggests their significant involvement in diverse biological processes in *V. vinifera* (Fig. 13). These results will provide directions for further research on the *VvJMJ* genes. Due to the data for gene expression calculations being sourced from online databases, we performed qRT-PCR analysis on a select few gene family members related to fruit development, MeJA, and SA treatments. We recommend conducting experimental verification to elucidate the precise functions of *VvJMJ* genes of interest to researchers.

## Conclusions

This study identified 23 *VvJMJ* genes in *V. vinifera* through bioinformatics analysis, which are distributed on 12 chromosomes of different densities and have been analyzed for their protein physicochemical properties. They were categorized into five subfamilies according to their phylogenetic relationship with *Arabidopsis* and *O. sativa*. The *JMJ* gene family was highly conserved between *Arabidopsis* and *V. vinifera*. Promoter analysis indicated that *VvJMJs* are potentially crucial in *V. vinifera* fruit growth and development, as well as in responses to light, hormones, and stress. Analysis of *VvJMJs* transcription levels across different *V. vinifera* varieties and developmental stages suggested diverse roles in fruit development, with distinct variety-specific and stage-specific traits. *VvJMJ18* and *VvJMJ16* demonstrated a decreasing trend during fruit ripening, while *VvJMJ23* was highly expressed in the Touch stage across six varieties. *VvJMJ4*, *VvJMJ12*, *VvJMJ13*, *VvJMJ2*1 may participated in the hormone response process to exogenous MeJA in *V. vinifera*; *VvJMJ4*, *VvJMJ6*, *VvJMJ15*, *VvJMJ17*, and *VvJMJ21* participated in the response to exogenous SA in *V. vinifera*. *VvJMJ1* and *VvJMJ13* may participate in the response to exogenous NAA in *V. vinifera*; *VvJMJ20* may participate in the response to exogenous ABA in *V. vinifera*. *VvJMJ13*, *VvJMJ15*, *VvJMJ20*, and *VvJMJ23* may participate in the response to ethylene. *VvJMJ1* and *VvJMJ10* could be associated with the *V. vinifera* photo stress response. *VvJMJ1*, *VvJMJ4*, *VvJMJ11*, *VvJMJ12*, *VvJMJ17*, *VvJMJ20*, *VvJMJ22*, *VvJMJ23* may participate in regulating the process of *V. vinifera* response to biotic stress. In summary, our results have laid a foundation for further studying the functions of *VvJMJ* genes and exploring the mechanisms of *V. vinifera* growth and development and fruit maturation.

## Supplementary Information

Supplementary Material 1.

Supplementary Material 2.

Supplementary Material 3.

Supplementary Material 4.

Supplementary Material 5.

Supplementary Material 6.

## Data Availability

The expression pattern of *VvJMJ* genes were obtained from SRA database in NCBI website. The study IDs were as follows: GSE62745, GSE62744, GSE150343, PRJNA845078, GSE129916 and PRJNA952825.

## Abbreviations

JmjC: Jumonji C
JMJs: Jumonji C domain
qRT-PCR: Quantitative real-time PCR
ABA: Abscisic Acid
MeJA: Methyl Jasmonate
NAA: 1-Naphthlcetic Acid
SA: Salicylic acid
ETH: Ethylene
